# Population kinetics of homoarginine and optimized supplementation for cardiovascular risk reduction

**DOI:** 10.1007/s00726-022-03169-x

**Published:** 2022-05-26

**Authors:** Christine J. Kleist, Chi-Un Choe, Dorothee Atzler, Mirjam Schönhoff, Rainer Böger, Edzard Schwedhelm, Sebastian G. Wicha

**Affiliations:** 1 Department of Clinical Pharmacy, Institute of Pharmacy, University of Hamburg, Bundesstraße 45, 20146 Hamburg, Germany; 2 Department of Neurology, University Medical Center Hamburg-Eppendorf, Hamburg, Germany; 3 Institute for Cardiovascular Prevention, Ludwig-Maximilians-Universität, Munich, Germany; 4 German Centre for Cardiovascular Research (DZHK), Partner Site Munich Heart Alliance, Munich, Germany; 5 Walther Straub Institute of Pharmacology and Toxicology, Ludwig-Maximilians-Universität, Munich, Germany; 6 Institute of Clinical Pharmacology and Toxicology, University Medical Center Hamburg-Eppendorf, Hamburg, Germany; 7 German Centre for Cardiovascular Research (DZHK), Partner Site Hamburg/Kiel/Lübeck, Hamburg, Germany

**Keywords:** Homoarginine, Population pharmacokinetics, Pharmacometrics, Kinetic, Amino acids, Cardiovascular disease

## Abstract

Homoarginine is an endogenous amino acid whose levels are reduced in patients with renal, cardio- and cerebrovascular disease. Moreover, low homoarginine concentrations independently predict morbidity and mortality in these patients. Besides endogenous synthesis, homoarginine is also a constituent of the human diet. The objective of the present study was to analyze the kinetics of orally supplemented homoarginine in human plasma by means of a pharmacometric approach. We developed a pharmacometric model to evaluate different dosing regimens, especially the regimen of 125 mg once weekly, based on a previous clinical study (*n* = 20). The model was adapted to account for differences in baseline homoarginine plasma concentrations between healthy and diseased individuals. A novel dosing regimen of 25 mg once daily led to higher attainment of homoarginine reference concentrations using clinical trial simulations. With 25 mg/day, the trough concentration of only 6% of the older and 3.8% of the younger population was predicted to be below the target concentration of 2.0–4.1 µmol/L. In synopsis, the new dosing regimen recapitulates the kinetics of homoarginine in healthy individuals optimally.

## Introduction

In 2013, two independent genome-wide association (GWA) studies uncovered that homoarginine plasma concentrations in humans are highly associated with single nucleotide polymorphisms (SNPs) in the l-arginine:glycine amidino transferase (*AGAT)* gene (Choe et al. 2013a; Kleber et al. 2013). Further studies identified AGAT as the responsible enzyme for homoarginine synthesis in vivo (Davids et al. 2012). AGAT is mainly expressed in the kidney and homoarginine is transported by cationic amino acid transporters into and out of cells and was found in all investigated tissues (Atzler et al. 2011; Choe et al. 2013b; Chafai et al. 2017; Taghikhani et al. 2019). The circulating plasma concentration of homoarginine in healthy humans shows considerable variation, i.e., the median plasma concentration of homoarginine was found to be 1.88 [25th and 75th percentile 1.47 and 2.41] µmol/L (Atzler et al. 2016a). Homoarginine plasma concentrations are dependent on sex, age and *AGAT* SNPs. In clinical studies, circulating homoarginine was evaluated in patients with kidney transplants, chronic kidney disease and patients suffering from cardio- or cerebrovascular disease with low concentrations being independently associated with disease phenotype and severity (Choe et al. 2013a; Atzler et al. 2016c; Kayacelebi et al. 2017). Moreover, low homoarginine plasma concentrations predicted mortality both in patients suffering from chronic kidney or heart disease, heart failure, or stroke as well as in the general population (Choe et al. 2013a; Ravani et al. 2013; Atzler et al. 2013, 2014, 2016b; Lew et al. 2017). Besides from endogenous synthesis by AGAT anabolism, homoarginine is a constituent of human diet (Rao et al. 1962; Bell 1962a, b) and no adverse effect of oral supplementation has been reported in short-term trials in healthy human (Atzler et al. 2016d; Schönhoff et al. 2018).

The objective of the present study was to analyze the kinetics of homoarginine in human plasma by means of a pharmacometric approach. This approach is common to describe the pharmacokinetics of drugs in a population (Owen and Fiedler-Kelly 2014). Therefore, measured plasma concentrations from all study participants are pooled and evaluated together. Inter-individual variability in the pharmacokinetic parameters is thereby considered to describe differences between the individuals and dissected from intra-individual variability in the pharmacokinetic profile. Covariates, such as age, weight or sex, are evaluated to explain parts of the inter-individual variability. For our study, we utilized this approach with the aim to describe the typical kinetic parameters, their inter-individual variability as well as the detection of covariates of these measures, of the endogenous amino acid homoarginine, which was administered as dietary supplementation. Moreover, the developed pharmacometric model was used to evaluate a previously used dosing regimen of 125 mg homoarginine once weekly and to propose a new dosing regimen for homoarginine supplementation, best mimicking the endogenous homoarginine levels of healthy individuals.

## Methods

### Human dataset

The dataset comprised anthropometric measures and concentration–time data of homoarginine plasma concentrations from a run-in phase and a double-blind, placebo-controlled crossover supplementation study (clinicaltrials.gov NCT02675660), study details and raw pharmacokinetic described elsewhere (Atzler et al. 2016d). In the run-in phase, 22 participants received a single oral dose of 125 mg homoarginine. In the placebo-controlled crossover study, the participants received 125 mg homoarginine or placebo once daily for 4 weeks. The study periods were separated by a washout phase of 4 weeks. EDTA plasma samples were drawn at time points 0, 0.25, 0.5, 1, 2, 4, 8, 24, 48, 72 and 120 h after the application of the single dose and after the last dose in each phase of the study. It was assumed that after 4 weeks of homoarginine supplementation, the plasma concentration reached the steady state. Due to discontinued intervention, two participants were excluded and homoarginine plasma concentrations were determined as previously described (Atzler et al. 2016d).

### Population kinetic analysis

For population kinetic modeling, NONMEM 7.4 (Icon Development Solutions, Ellicott City, Maryland, USA) was used and executed via PsN (V4.7.0) (Lindbom et al. 2005). Inter-individual variability was implemented on the structural kinetic parameters as follows:

$${P}_{k,i}={\theta }_{k}\times {e}^{{\eta }_{k,i}}$$

where *P*_*k,i*_ represents the estimated *k*th kinetic parameter for the *i*th individual calculated from the population kinetic parameter *θ*_*κ*_ of the typical patient, while *η*_*κ,i*_ represents the individual deviation from the typical kinetic parameter assuming log-normal distribution. The residual variability in an individual patient at each time point, that is, the difference between individual model-predicted (*Y*_PRED,*i,j*_) and the observed homoarginine concentration (*Y*_OBS,*i,j*_) for the *i*th subject at the *j*th time point, was estimated by a proportional (*ε*_*ρ*,*i,j*_), additive (*ε*_*α*,*i,j*_) or combined residual variability model:

$${Y}_{\mathrm{OBS},i,j}={Y}_{\mathrm{PRED},i,j}\times \left(1+{\varepsilon }_{p,i,j}\right)+{\varepsilon }_{a,i,j}.$$

For the models with implemented baseline (BSL), the individual model-predicted concentration (*Y*_PRED,*i,j*_) was the sum of the predicted individual baseline concentrations and the individual specific model predictions (*F*):

$${Y}_{\mathrm{PRED}, i, j}= F+\mathrm{BSL}.$$

One- and two-compartment kinetic models with first-order oral absorption, first-order disposition and elimination processes with or without BSL were evaluated. Inter-individual variability (IIV) was assessed on all structural parameters.

To explain the observed IIV of the structural kinetic parameters, sex as categorical covariate, patient total body weight, body height, ideal body weight and age as continuous covariates were assessed on clearance (CL), central volume of distribution (*V*_2_), BSL and peripheral volume of distribution (*V*_3_). Additionally, the estimated glomerular filtration rate [estimated by (i) Cockcroft-Gault (Cockcroft and Gault 1976), (ii) MDRD (Levey et al. 2003) and (iii) CKD-EPI (Levey et al. 2009)] was assessed as a covariate for CL (Table 1). These covariates showed a potential relationship with the measured homoarginine plasma concentrations in exploratory graphical analysis. Further, total body fat, serum creatinine, sodium, potassium, chloride, osmolarity, urea, leucocytes, erythrocytes, hemoglobin, hematocrit, MCH, MCHC, MCV, thrombocytes, AST, ALT, glucose and alkaline phosphatase were assessed in the exploratory data analysis.

**Table 1:** Distribution of covariates in the population used to develop the population kinetic model of homoarginine (*n* = 20 participants)

Covariate	Median	min	Max
Weight [kg]	67.65	48.75	108.1
Height [cm]	172.3	153.7	199.5
Ideal body weight [kg]	63.97	47.05	93.23
Age [years]	28	21	61
eGFR (Cockcroft–Gault) [mL/min]	117.8	65.77	168.3
eGFR (MDRD) [mL/min]	105.4	60.99	146.3
eGFR (CKD-EPI) [mL/min]	111.5	65.24	145.9
Sex	45% male (*n* = 9)	55% female (*n* = 11)	

Covariate effects were tested using standard forward inclusion and backward elimination with a forward significance level of 0.05 (drop in objective function values (dOFV) > 3.84) and a backward significance level of 0.01 (dOFV > 6.63) employing the likelihood ratio test. For non-nested models, in case of competing models, the Akaike Information criterion (AIC), was used for model selection. For the forward inclusion, all covariates were independently tested, by including one covariate in to the final structural model at a time. Guided by the highest dOFV, the strongest covariate was implemented. In addition, covariates not correlated to the included one were implemented analog to the inclusion of the first one. In each step, the strongest covariate was included until no covariate was significant anymore. For the backward elimination again, one by one was excluded applying the stricter significance criterion.

For the selection of the model which described the kinetics of homoarginine best, goodness-of-fit plots (GOF) of population and individual prediction vs. observed concentrations were used as graphical model diagnostic criteria. In addition, visual predictive checks (VPCs) (*n* = 1000) were performed and conditionally weighted residuals (CWRES) as well as normalized prediction distribution errors (NPDE) were inspected.

To evaluate parameter uncertainty, a nonparametric bootstrap analysis with *n* = 1000 samples was performed.

### Simulations

The clinical dataset included a younger (21–34 years, *n* = 14) and older (43–61 years, *n* = 6) population. To avoid extrapolation, two simulation datasets with *n* = 10,000 virtual individuals each were built using the population means of age and height of these two clinical subpopulations for creating randomly distributed covariates [age (young): mean = 26 years, standard deviation (SD) = 0.08; age (old): mean = 52 years, SD = 0.09; height: mean = 175 cm, SD = 0.045]. The body height was used to calculate the ideal body weight.

A phase 2 trial in patients suffering from acute ischemic stroke has been recently initiated supplementing individuals with placebo or 125 mg homoarginine once weekly that are screened with the rapid test for low homoarginine (clinicaltrials.gov NCT03692234). To identify suitable regimens for supplementation in patients with low homoarginine levels, the model was adapted for low endogenous homoarginine plasma concentrations. Therefore, the population mean of BSL was adjusted so that 95% of the patients displayed homoarginine plasma concentrations below 2 µmol/L. The concentration of 2.0 µmol/L was chosen in line with a rapid test, which should identify patients at increased cardiovascular risk associated with a low homoarginine concentration. The BSL was set to 1.29 µmol/L (IIV: 28.8%) mimicking the clinical population with low homoarginine concentrations.

The final model with the adjusted BSL value was used to perform simulations with various dosing regimens. The evaluated dosing regimen comprised once daily dosing of 1.25 mg, 2.5 mg, 5 mg, 10 mg, 12.5 mg, 15 mg, 20 mg, 25 mg, 30 mg, 40 mg, 50 mg and weekly dosing of 125 mg.

The target concentration range for supplementation with homoarginine was defined from 2.0 to 4.1 µmol/L. The maximum was based on the maximum reference concentration in healthy male subjects (Atzler et al. 2016a). The minimum concentration was defined as 2.0 µmol/L which is associated with an increased cardiovascular risk when concentrations are below that threshold value (Cordts 2018).

The simulation results were presented graphically. The time in the target concentration range was assessed. The impact of patient covariates on the target concentrations and the time in the target concentration range was explored to assess the potential value of covariate-based stratified dosing regimens.

## Results

### Population kinetic analysis

The best structural model was a two-compartment model with BSL. This model was superior to a one-compartment without BSL (dAIC = − 758.949) and with BSL (dAIC = − 97.259) and to a two-compartment without BSL (dAIC = -695.266).

IIV was supported on CL (dOFV = − 115.206), *V*_2_ (dOFV = − 121.862), *V*_3_ (dOFV = − 3.717) and BSL (dOFV = − 290.831) with the reduction of the OFV compared to the final base model. Shrinkage of the individual parameters toward the population mean was low (≤ 4%) for CL, *V*_2_ and BSL, and high (45%) for *V*_3_ indicating that only few patients supported the estimation of the IIV on *V*_3_.

A separate residual variability model was implemented for BSL and the dose-related homoarginine plasma concentrations. For the BSL data, a combined model (proportional and additive) was chosen. It was superior to a sole proportional error model (dOFV = − 17.087) and a sole additive error model (dOFV = − 1.654). For the homoarginine dose-related plasma concentrations, the additive error component tended to zero, so a sole proportional residual variability model was superior (dOFV = 0).

Ideal body weight was found as a covariate on CL (dOFV = − 22.518) and *V*_3_ (dOFV = − 6.881) and reduced the unexplained inter-individual variability for Cl from 27.9 to 14.2% and for *V*_3_ from 26.9 to 17.8%. Age was found as covariate for *V*_2_ (dOFV = − 18.634) resulting in a reduction in IIV on *V*_2_ from 95.9 to 50%. As the IIV on *V*_3_ was not stable in the bootstrap analysis and the improvement of the model fit was only minor after covariate inclusion (dOFV = − 1.431), the IIV on *V*_3_ was not considered in the final model anymore.

**Fig. 1:**
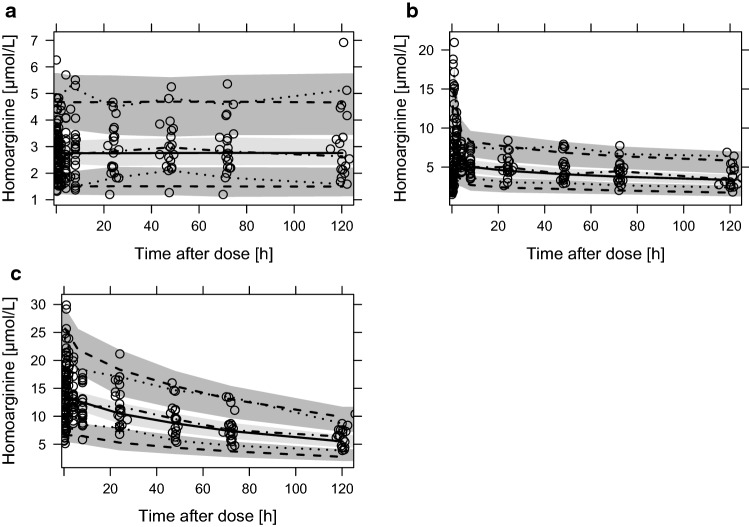
Visual predictive check stratified on baseline (**a**), single dose (**b**) and dose in steady state (**c**) for the final kinetic model. Observed homoarginine concentrations (points) and observed median (dot dashed line) with 5th to 95th percentile (dotted lines) compared to predicted median (solid line) with 5th to 95th percentile (dashed lines) and 95% confidence interval (shaded areas)

**Fig. 2:**
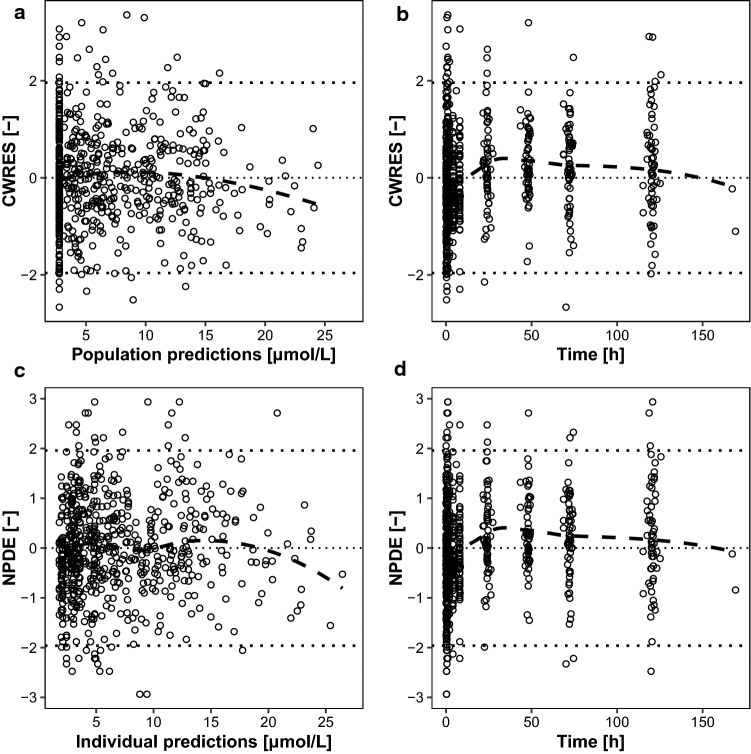
Conditionally weighted residuals (CWRES) versus individual population predictions (**a**) and time after dose (**b**) with LOESS smoothing curve (dashed line). Normalized prediction distribution errors (NPDE) versus individual population predictions (**c**) and time (**d**) with LOESS smoothing curve (dashed line)

The final population kinetic parameters are presented in Table 2. The visual predictive check indicated a good prediction for the homoarginine concentration–time profiles in plasma (Fig. 1). The analysis of CWRES and NPDE also indicated a good model fit (Fig. 2). The bootstrap indicated that the parameters were estimated with precisely (Table 2).

**Table 2:** Typical kinetic parameters (*θ*), unexplained inter-individual variability estimated as variance (*ω*^2^), and residual variability estimated as variance (*σ*^2^) obtained from the pharmacometric analysis

Kinetic parameter	Estimate	95% CI	RSE (%)
Clearance, CL = *θ*_1_ × (IBW/75)^*θ*_7_ [L/h]			
*θ*_1_	3.67	3.25, 4.26	6.5
*ω*_1_^2^	0.0225 (*ω*_1:_ 15.1% CV)	0.00322, 0.0419	24.9
Central volume of distribution, *V*_2_ = *θ*_2_ × (AGE/29)^*θ*_8_ [L]			
*θ*_2_	69.5	54.5, 84.8	11.1
*ω*_2_^2^	0.21 (*ω*_2:_ 48.3% CV)	0.0342, 0.338	18.3
Peripheral volume of distribution, * V*_3_ = *θ*_3_ × (IBW/75)^*θ*_9_ [L]			
*θ*_3_	235	177, 312	12.6
First-order absorption rate constant, *k*_a_ = *θ*_4_ [h^−1^]			
*θ*_4_	0.924	0.722, 1.14	11.1
Distribution clearance, *Q* = *θ*_5_ [L/h]			
*θ*_5_	28.2	24.7, 33.2	6.7
Baseline, BSL = *θ*_6_ [µmol/L]			
*θ*_6_	2.77	2.44, 3.15	6.5
*ω*_3_^2^	0.0799 (*ω*_3:_ 28.8% CV)	0.0361, 0.123	13.6
Covariate ideal body weight (IBW) on CL = *θ*_7_			
*θ*_7_	1.4	0.897, 1.99	18
Covariate age on * V*_2_ = *θ*_8_			
*θ*_8_	− 1.86	− 2.54, − 1.20	15.4
Covariate ideal body weight (IBW) on *V*_3_ = *θ*_9_			
*θ*_9_	1.27	0.225, 2.43	35.3
Residual variability model			
*σ*_proportional,baseline_	15.5% CV	0.124, 0.188	10.8
*σ*_additive,baseline_	0.267 µmol/L	Fixed^a^	53.2
*σ*_proportional_	25.6% CV	0.237, 0.274	3.8

*IBW* ideal body weight, *CV* coefficient of variation, *RSE* relative standard error (reported on standard deviation (SD) scale for variability parameters), *CI* 95% confidence interval determined by a non-parametric bootstrap analysis (*n* = 1000)

The transformation of *ω*^2^ to coefficient of variation was calculated by $\sqrt{\left({e}^{{\omega }^{2}}-1\right)}\times 100 \%$

^a^Fixed to final estimate during bootstrap to increase model stability

The influence of the covariates manifested itself in the empirical Bayesian estimates in the reduction of *V*_2_ with increasing age (*V*_2_: 145–13.8 L, 22–61 years) and increasing CL and *V*_3_ with increasing ideal body weight (CL: 1.65–5.24 L/h, *V*_3_: 129.82–309.21 L, 47.07–93.23 kg).

### Simulations

First, a currently used standard dosing regimen for supplementation consisting of 125 mg oral homoarginine administered once per week was simulated. The peak concentration yielded by this dosing regimen was about 2.5 times or about 1.5 times higher, in the older or in the young population, respectively, when compared to the maximum physiological target concentration (*c*_peak, median, old_ = 10.42 µmol/L, *c*_peak, median, young_ = 6.13 µmol/L). The trough concentration was predicted to 1.64 µmol/L (old population) and 1.76 µmol/L (young population) in median and thus below the physiological target of 2.0 µmol/L.

**Fig. 3:**
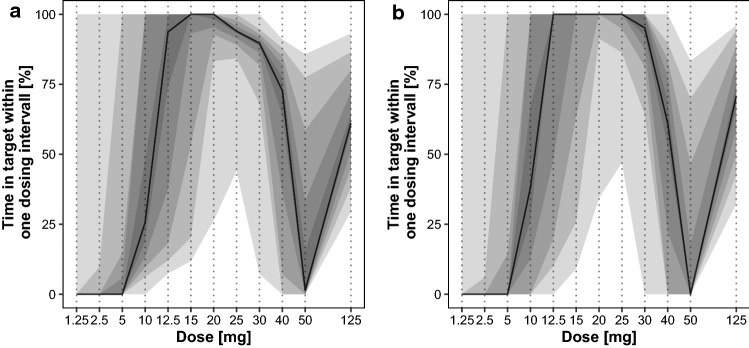
Percentage of time in target within one dosing interval in steady state [dosing interval: 24 h, for the dose from 1.25 to 50 mg and reference 125 mg weekly, i.e., 168 h (right apart)] for the older (**a**) and young population (**b**). The population median is given by the solid line and variability is illustrated by shaded areas as 5th to 95th percentile in 10-percentile steps

For the once-weekly 125 mg regimen, only 8.99% of the older population and 22.1% of the younger population achieved > 90% time in target range in steady state. The median time in the target range was 61.1% (old) or 70.7% (young) (Fig. 3, Table 3).

**Table 3:** Assessment of the weight dependence of various homoarginine dosing regimens; given percentage indicate the number of subjects laying > 90% of time in target range

Population	Old	Old	Old	Young	Young	Young
Dosing regimen	60 kg ± 0.5 kg	80 kg ± 0.5 kg	All	60 kg ± 0.5 kg	80 kg ± 0.5 kg	all
12.5 mg q 24 h	67.5%	37.2%	50.4%	71.5%	38.4%	54.3%
15 mg q 24 h	80.9%	47.0%	63.7%	84.2%	47.6%	67.6%
20 mg q 24 h	87.6%	70.7%	79.6%	92.6%	76.2%	84.1%
25 mg q 24 h	60.4%	79.3%	74.1%	73.8%	84.1%	82.2%
125 mg q 168 h	0.33%	20.1%	9.0%	17.4%	24.4%	22.1%

To increase the time in the target range, 24 h dosing intervals and lower daily doses were explored to avoid both high supraphysiological peak concentrations and underexposure. The median time in the target range in steady state was highest for daily doses of 12.5–25 mg. The highest fraction of patients being in the target range was achieved at a dose of 20 mg daily with 79.6% of the older, and 84.1% of the young patients achieving > 90% time in the target range (Fig. 3).

The impact of covariates on homoarginine kinetics for the 20 mg q 24 h regimen was investigated: for age, a difference of the predicted homoarginine concentration–time profiles between the group of young (median age: 26 years) and older subjects (median age: 52 years) was detected. The concentration achieved one hour after dosing was lower (*c*_1h, median, old_ = 3.77 µmol/L, *c*_1h, median, young_ = 3.11 µmol/L) compared to young subjects due to the decrease of *V*_2_ with increasing age.

The impact of weight on target time for the different dosing tiers ranging from 12.5 to 125 mg is presented in Table 3.

Overall, doses from 20 to 25 mg q 24 h resulted in the highest fraction of the population reaching > 90% time in the target concentration range.

## Discussion

In the present study, a population kinetic model of homoarginine supplementation in human subjects was developed. A covariate analysis was performed and ideal body weight and age emerged as significant covariates of homoarginine kinetics.

The covariate relation of age and central volume of distribution is an inverse correlation. The increase of age is physiologically correlated with decreasing body water, which is described as volume of distribution in the model. The second covariate relation indicates the increase of the clearance in relation to the increasing ideal body weight. This could be explained by the bigger size of organs in heavier patients. Ideal body weight (IBW) was superior as a covariate on clearance compared to the real body weight. The dependence of homoarginine plasma concentrations on body weight is in line with previous studies in human and mice. Increasing body weight correlated with lower homoarginine plasma concentrations in mice fed with a high-fat diet or patients undergoing bariatric surgery (May et al. 2015; Stockebrand et al. 2015).

The developed model describes the population kinetics of healthy subjects. Therefore, homoarginine was in the target range without supplementation. As the objective of the kinetic analysis was the identification of dosing regimens for patients at low homoarginine concentrations, the model had to be adjusted by modification of the baseline value. The homoarginine predictions obtained from the modified model with the lowered baseline matched subjects at cardiovascular risk displaying decreased homoarginine concentrations.

A currently investigated weekly dose for supplementation of 125 mg homoarginine in stroke patients (clinicaltrials.gov NCT03692234) was compared to alternative daily dosing regimens exploring doses of 1.25–50 mg with respect to achieving homoarginine concentrations similar to the healthy population. As low homoarginine has been found in renal and cardio- or cerebrovascular disease patients (Choe et al. 2013a; Atzler et al. 2016c; Kayacelebi et al. 2017) and low homoarginine plasma concentrations predict morbidity and mortality (Choe et al. 2013a; Ravani et al. 2013; Atzler et al. 2013, 2016b), it seems rationale to avoid underexposure. Compared to the dosing interval of 125 mg once weekly, the time in target could be increased with daily dosing and high unphysiological peak concentrations as well as low trough values could be avoided. Daily homoarginine supplementations of 20 or 25 mg yielded highest target attainment, with 79.6% vs 74.1% (old population) and 84.1% vs 82.2% (young population) of the population laying > 90% time in target, for 20 mg and 25 mg respectively. With higher doses, the maximal plasma concentrations are increasing. Therefore, more patients were exceeding the upper target concentration and laid outside of the target range with the 25 mg dosing regimen. However, a dose of 25 mg/day would be favorable, because compared to the dose of 20 mg, few subjects were predicted to display homoarginine plasma concentrations below the target range. At 25 mg/day, the trough concentration of only 6% of the older and 3.8% of the younger population was predicted to be below the target concentration.

As the covariates age and IBW displayed an impact on the kinetics of homoarginine, a dose adaption was taken into consideration. Hence, the target attainment was assessed in different subpopulations, stratified by age and weight. Especially in the promising dosing regimen of 20 mg and 25 mg q 24 h, the impact of the covariates in the target attainment predictions was minor. The differences between the investigated subpopulations are below 20% and therefore likely not clinically relevant.

Few limitations of the analysis have to be acknowledged. The most important point is the extrapolation of data from healthy individuals to patients. Further studies are required to validate this extrapolation in patients to ensure similar homoarginine metabolism. Indeed, diseased patients might suffer from reduced renal function and thus prolonged elimination of homoarginine. Nonetheless, renal function was not identified as a covariate in the present population. Moreover, we assumed that the homoarginine kinetics reached a steady state after 4 weeks of daily dosing, which also requires verification in clinical studies. Future studies should also evaluate if other food constituents or supplements such as creatine, which share common metabolic pathways may impact the kinetics of homoarginine.

## Conclusion

In the present study, we analyzed the kinetics of homoarginine in human plasma by means of a pharmacometric approach. The analysis identified age and central volume of distribution as covariates of these measures. From the developed pharmacometric model, a new dosing regimen for homoarginine supplementation of 25 mg once daily is proposed. However, it has to be evaluated in clinical trials, if this dosing regimen in patients mimics the endogenous homoarginine levels of healthy individuals best.
